# Counting the Acid Sites in a Commercial ZSM-5 Zeolite Catalyst

**DOI:** 10.1021/acsphyschemau.2c00040

**Published:** 2022-11-01

**Authors:** Andrea Zachariou, Alexander P. Hawkins, Russell F. Howe, Janet M. S. Skakle, Nathan Barrow, Paul Collier, Daniel W. Nye, Ronald I. Smith, Gavin B. G. Stenning, Stewart F. Parker, David Lennon

**Affiliations:** † School of Chemistry, University of Glasgow, Joseph Black Building, GlasgowG12 8QQ, U.K.; ‡ UK Catalysis Hub, Research Complex at Harwell, STFC Rutherford Appleton Laboratory, ChiltonOX11 0FA, Oxon, U.K.; § Department of Chemistry, University of Aberdeen, AberdeenAB24 3UE, U.K.; ∥ Department of Physics, University of Aberdeen, AberdeenAB24 3UE, U.K.; ⊥ Johnson Matthey Technology Centre, Blounts Court, Sonning Common, Reading, BerkshireRG4 9NH, U.K.; # ISIS Facility, STFC Rutherford Appleton Laboratory, ChiltonOX11 0QX, Oxon, U.K.

**Keywords:** HZSM-5, inelastic neutron scattering (INS), DRIFTS, NMR, SEM, ammonia chemisorption

## Abstract

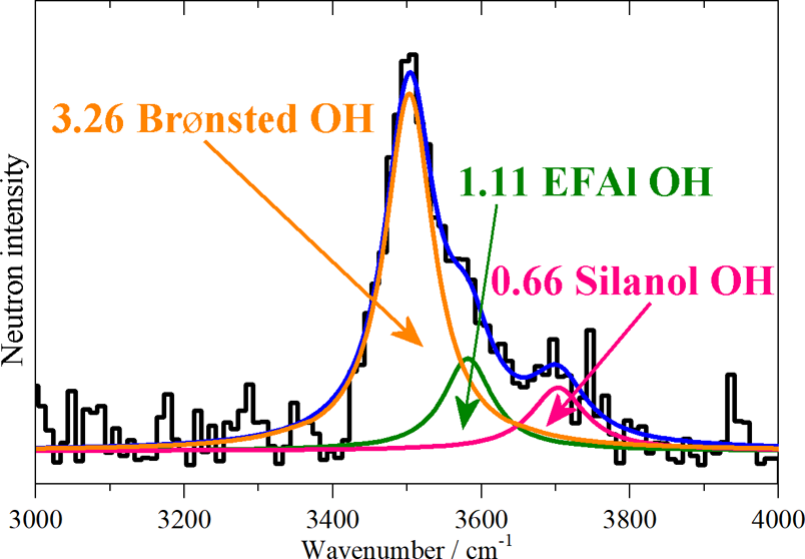

This work investigates the acid sites in a commercial ZSM-5 zeolite catalyst by a combination of spectroscopic and physical methods. The Brønsted acid sites in such catalysts are associated with the aluminum substituted into the zeolite lattice, which may not be identical to the total aluminum content of the zeolite. Inelastic neutron scattering spectroscopy (INS) directly quantifies the concentrations of Brønsted acid protons, silanol groups, and hydroxyl groups associated with extra-framework aluminum species. The INS measurements show that ∼50% of the total aluminum content of this particular zeolite is extra framework, a conclusion supported by solid-state NMR and ammonia temperature-programmed desorption (TPD) measurements. Evidence for the presence of extra-framework aluminum oxide species is also seen in neutron powder diffraction data from proton- and deuterium-exchanged samples. The differences between results from the different analytical methods are discussed, and the novelty of direct proton counting by INS in this typical commercial catalyst is emphasized.

## 1 Introduction

Zeolites are an important class of micro- and mesoporous materials. While they occur naturally, most commercial applications make use of synthetic materials.^1,2^ One of the most important of these is the MFI framework group, exemplified by ZSM-5. This was invented by the Mobil Oil Company in 1975^3,4^ and has found widespread application in industry for reactions ranging from hydrocracking^5^ to toluene disproportionation (to generate benzene and xylenes)^6^ to methanol-to-hydrocarbons (MTH)^7^ (which includes methanol-to-olefins (MTO)^8^ and methanol-to-gasoline (MTG)^9^) chemistries.

Chemically, ZSM-5 is an aluminosilicate with the formula: A_*n*_Al_*n*_Si_96–*n*_O_192_·16H_2_O (0 ≤ *n* < 27), where A is a charge balancing cation. This may be an alkali metal (usually Na^+^), but the industrially significant form has A = H. This is required because the presence of Al^3+^ in the otherwise all-Si^4+^ structure results in a charge imbalance. In the proton form, the hydrogen resides on the bridging oxygen atom of the Al–O–Si linkage. This generates a Brønsted acid site that is responsible for the catalytic activity of the zeolite.

Crucial to the performance of the catalyst is the Si:Al ratio and hence the number (and location) of the Brønsted acid sites. In this paper, we present an in-depth characterization of a commercial HZSM-5 zeolite, from the macroscopic to the atomic scale. We highlight some of the issues that can arise in the description of the composition and present a novel method to determine the number, and type, of the hydroxyls present.

## 2 Experimental Methods

### 2.1 Materials

The HZSM-5 zeolite was provided in powder form by Johnson Matthey plc. Prior to use, the zeolite was calcined at 773 K for 12 h in static air to remove the residual template. The catalyst was then dried at 623 K under flowing He (100 mL min^–1^) in a previously described rig.^10^ A deuterated sample was prepared by passing a saturated stream of D_2_O in helium through the sample using a sealable container and a bubbler arrangement. The sample was heated to 573 K during the deuteration process, this temperature being high enough to promote the exchange of the Brønsted acid sites in the zeolite with deuterium from the heavy water, without being high enough to cause significant steam de-alumination of the zeolite framework.^11^ The deuteration process was continued for 5 h, and the sample was then dried as before.

### 2.2 Scanning Electron Microscopy (SEM)

A sample of HZSM-5 was examined by SEM on a Zeiss SEM300.

### 2.3 Surface Area and Pore Volume Measurements

Surface area analysis was performed using a Quantachrome Quadrasorb EVO/SI gas adsorption instrument. Samples (0.15 g) of the materials for analysis were degassed to <2.67 × 10^–2^ mbar (20 mTorr) at 523 K, and gas adsorption and desorption isotherms were collected across a relative pressure (*P*/*P*_0_) range of 5 × 10^–4^–0.99 using liquid nitrogen as the coolant and N_2_ as the adsorbent gas. Isotherm analysis was carried out using the Brunauer–Emmett–Teller (BET) method using the software supplied with the instrument. Sample microporosity levels were estimated using the *t*-plot method of de Boer.^12^ The measurements were repeated three times to allow an estimation of the degree of accuracy in the measurement.

### 2.4 X-ray Fluorescence (XRF)

A PANalytical Epsilon3 XL energy-dispersive instrument was used to measure the XRF data. The in-built software was used for quantitative analysis (labeled as analysis 1). XRF data was also supplied by the catalyst manufacturer (labeled as analysis 2).

### 2.5 Inelastic Neutron Scattering (INS) Spectroscopy

The INS experiments used TOSCA^13^ and MAPS^14,15^ spectrometers at the ISIS Pulsed Neutron and Muon Source (Oxfordshire, UK). The instruments are complementary: TOSCA provides good resolution spectra over the 0–2000 cm^–1^ range, while MAPS provides access to the C–H and O–H stretching regions (2000–4000 cm^–1^). The MAPS spectra were collected using the A-chopper package with fixed incident energies of 5244 cm^–1^ (600 Hz) and 2017 cm^–1^ (400 Hz). The INS spectra were recorded below 20 K to minimize the Debye–Waller factor. The MAPS spectrometer was calibrated using a series of brucite (Mg(OH)_2_) samples of different weights (53, 154, 320, and 620 mg). For the pyridine measurements, the zeolite was dosed at 373 K for 1 h with pyridine using a Dreschel bottle, which was kept at 294 K, with a helium carrier gas (100 mL min^–1^). At the end of the hour, the sample was flushed with helium for 10 min. For measurements on MAPS, the samples were transferred into an aluminum can, before being transferred back into the gas flow cells to desorb the pyridine. Pyridine desorption was carried out at 523 K under helium flow. The desorbed samples were then transferred back into their aluminum cans for the final MAPS measurements to take place. For measurements on TOSCA, the procedure was the same except that because the flow cell is compatible with TOSCA, no sample transfers were done.

### 2.6 Diffuse Reflectance Infrared Spectroscopy (DRIFTS)

The DRIFTS experiments used an Agilent Carey 680 Fourier-transform infrared spectroscopy (FTIR) spectrometer with a liquid nitrogen-cooled MCT detector and a Harrick Praying Mantis DRIFTS environmental chamber accessory; 64 scans per spectrum were collected at a resolution of 4 cm^–1^. Samples were dried at 623 K (ramp rate of 5 K min^–1^) under an N_2_ flow of 50 mL min^–1^ from liquid nitrogen boil-off, as we have found that this has the lowest water levels we can achieve. Spectra were collected every 20 K with the final spectrum collected after 30 min at 350 K.

### 2.7 Solid-State NMR (ss-NMR) Spectroscopy

The ss-NMR spectra were acquired at a static magnetic field strength of 14.1 T (*v*_0_ (^1^H) = 600 MHz) on a Bruker Avance Neo console using TopSpin 4.0 software. For ^29^Si, the probe was tuned to 119.23 MHz and referenced to kaolinite at −91.2 ppm. ^27^Al spectra were recorded at 104.2 MHz using a one-pulse Bloch decay with a 0.5 μs pulse width (π/20) and a 5 s pulse delay. All samples were measured with 500 acquisitions to allow comparison of signal-to-noise ratios. The chemical shifts were externally referenced to a kaolin standard (−2.5 ppm relative to [Al(H_2_O)_6_]^3+^). The samples were dried at 383 K before analysis. The samples were packed into zirconia MAS rotors with Kel-F caps; sample masses before and after weighing were provided. The rotors were spun using room-temperature purified compressed air. ss-NMR spectra were recorded at room temperature.

### 2.8 Powder Diffraction

Neutron powder diffraction (NPD) was carried out at room temperature on the POLARIS diffractometer at ISIS.^16^ Samples of the dried protonated and deuterated ZSM-5 were loaded into 8 mm diameter indium wire-sealed vanadium sample cans, sealed using indium wire, in an argon glovebox. The crystal structures were refined using GSAS-II^17^ initially using the highest-resolution, backscattering data at 2θ = 145°, then adding in the 2θ = 90° data set. This was done for both the **P**21̅*n* monoclinic structure of Koningsfeld et al.^18^ and the *Pnma* orthorhombic structure of Artioli et al.^19^ Initially, the instrumental parameters, peak widths, and background, together with the unit cell, were refined. For both data sets (H and D), the background was fitted using a Chebyschev function with 12 parameters given its shape—a higher number of parameters led to some level of instability in the refinement due to the rather undulating nature of the background. The time-of-flight limit was 4000–19 900 μs in both cases—inclusion of lower time-of-flight data did not improve the quality of the structural model and led to extremely long refinement times.

Powder X-ray diffractograms (XRD) were measured with a Rigaku Smartlab with a 9 kW source, Cu Kα anode with Ge(220) monochromator. The scan speed was 3° min^–1^, with the high-resolution spectra being performed at 1° min^–1^, with 6 rpm sample rotation. The in situ low-temperature measurements were collected using an Oxford Cryosystems Phenix stage. Measurements were taken between 13 and 300 K in 15 K steps, with a ramp rate of 6 K min^–1^.

### 2.9 Ammonia Temperature-Programmed Desorption (TPD)

TPD experiments were carried out using a Quantachrome ChemBET Pulsar instrument equipped with a thermal conductivity detector (TCD). Samples were dried at 623 K under flowing helium (15 mL min^–1^) then cooled to 373 K and saturated with ammonia by passing 10% NH_3_ in He (15 mL min^–1^) through the sample for 15 min. The sample was returned to helium flow and purged for 2 h at the same temperature; these conditions are reported to remove any physisorbed ammonia from the zeolite pore network leaving only molecules chemisorbed to Lewis silanol or Brønsted acid sites.^20^ Desorption was then carried out from 373 to 973 K with a heating rate of 5 K min^–1^ and a 30 min hold at the highest temperature to ensure full removal of all ammonia. The response of the TCD was normalized against that of ammonia injections of a known quantity to enable the determination of the volume of chemisorbed ammonia in each sample.

## 3 Results

As stated in Section 1, we investigate the materials from the macroscopic scale to the atomic scale. We first consider the morphology and composition of the sample by SEM, XRF, ss-NMR, and BET, and then investigate the average structure by neutron and X-ray diffraction. Finally, we characterize the hydroxyls, and hence the acid sites, by ammonia TPD and DRIFTS and INS spectroscopies.

### 3.1 Morphology and Composition

**Figure 1:**
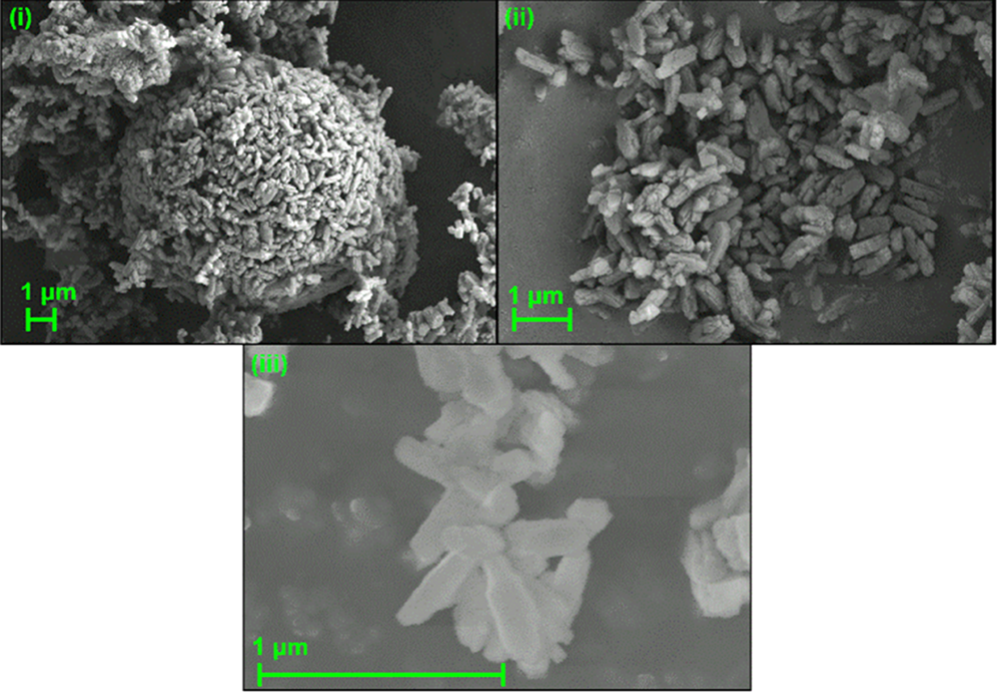
SEM images of HZSM-5 collected at (i) ×5000, (ii) ×12 000, and (iii) ×75 000 magnification.

The crystallite size is important to the catalytic activity of the zeolite because of how it affects the internal volume available for diffusion and the relative proportions of interior and surface acid sites. Zeolite crystals are usually large enough that attempting to size them by Scherrer analysis of the peak broadening in X-ray data does not produce useful results and crystallite sizing is therefore usually done by examination of the material using SEM. Figure 1 presents a representative sample of the images produced. Analysis of the structures in these images revealed that crystal sizes were in the 0.2–1.0 μm range with typical crystallite dimensions being 0.5 μm × 0.1 μm × 0.1 μm. It can also be seen that the individual crystallites have a tendency to stick together into larger agglomerations.

Determining the aluminum content of the zeolite is vital, as it determines the number of Brønsted acid sites. However, there is a possible confusion in the literature that expresses the aluminum content as either the SiO_2_:Al_2_O_3_ ratio (SAR) or the atomic Si:Al ratio. SAR is relevant for catalyst preparative purposes, whereas the term Si:Al is more convenient when describing acid site densities for a particular catalyst formulation. Thus, the academic literature on performance characteristics of zeolites applied to specific reactions tends to use the Si:Al ratio to describe the zeolite. Table 1 presents the XRF analysis of this sample by two different benchtop instruments at two different institutions. Both agree on a Si:Al ratio of approximately 16. Note the difference between the SAR and the Si:Al values.

**Table 1:** XRF Analysis of ZSM-5

	SiO_2_/wt %	Al_2_O_3_/wt %	SiO_2_:Al_2_O_3_	Si:Al
analysis 1	95.10	4.85	33.3:1	16.6:1
analysis 2	95.00	4.96	32.5:1	16.3:1

An alternative method for determining framework Al content is via ^29^Si-ssNMR. As described by Fyfe et al.,^21^ the ^29^Si NMR spectrum of a zeolite shows different chemical shifts for silicon bonded through oxygen to different numbers of next nearest-neighbor aluminum atoms: i.e., Si(OSi)_4_, Si(OSi)_3_(OAl), Si(OSi)_2_(OAl)_2_, etc. For high silica zeolites, such as ZSM-5, it is only the first two of these that contribute to the spectrum. A signal at the same chemical shift as Si(OSi)_2_(OAl)_2_ is also seen because of silanol groups (Si(OSi)_3_(OH)). From the relative intensities of these signals, it is possible to estimate the framework aluminum content of the zeolite, assuming Lowenstein’s rule^22^ (no Al–O–Al linkages) is followed.
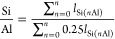
1

Equation 1 is used to determine the Si:Al ratio of a zeolite with the assumption that the zeolite follows Lowenstein’s rule.^22^ Here, *n* is the number of Al present in each of the peaks and *l* is the integrated area of the Gaussian used for fitting the peak. This equation is adapted from ref (21).

**Figure 2:**
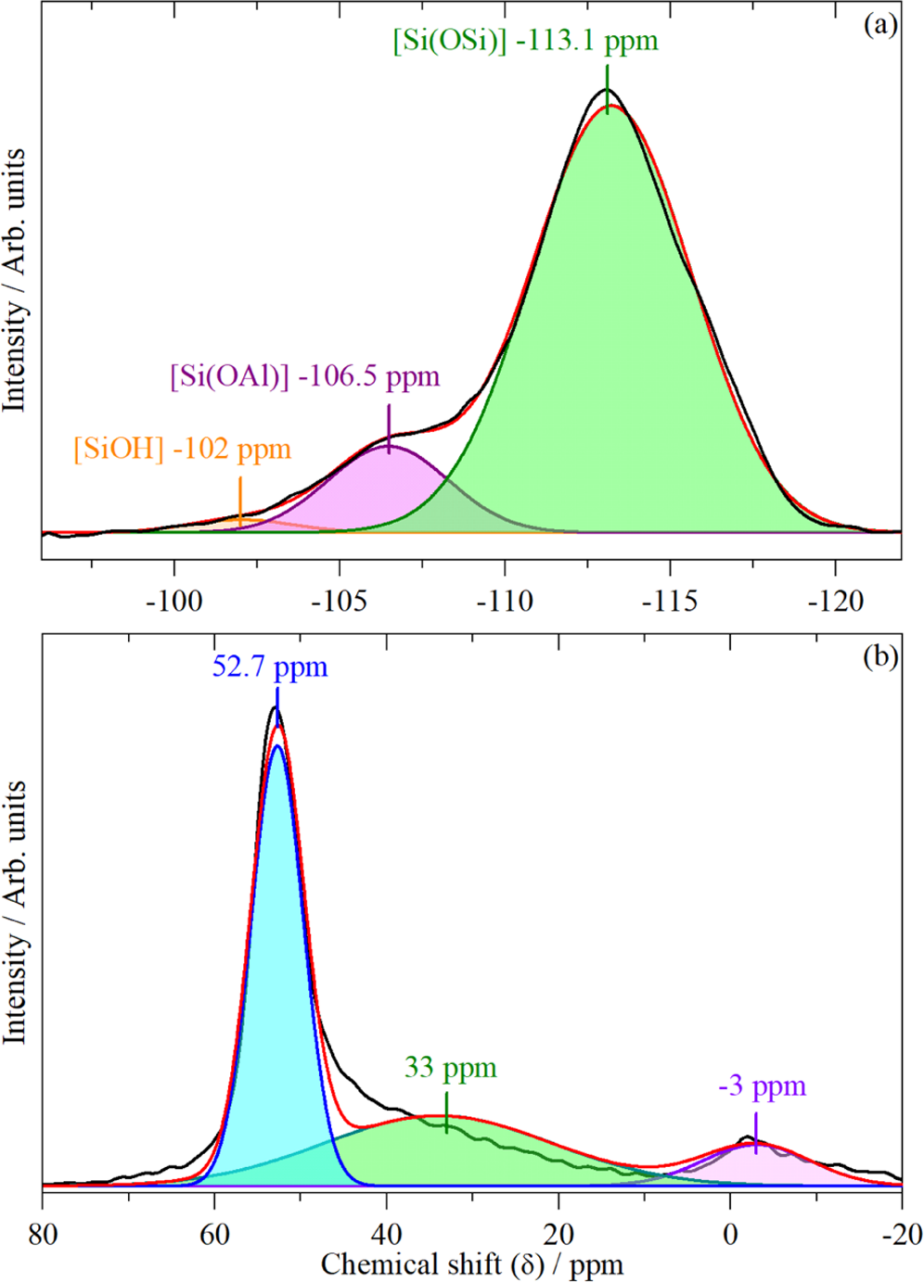
(a) Peak fitting of ^29^Si-ssNMR spectrum of ZSM-5. Three peaks are used to simulate the experimental spectrum (black): Si atoms with only Si atom neighbors (green), silicon atoms with one aluminum atom attached to it (purple), and silanols (orange). The total fit (red) is also shown. (b) Peak fitting of the ^27^Al-ssNMR spectrum of ZSM-5. Key: experimental spectrum (black), total fit (red), tetrahedral Al (blue), octahedral extra framework Al (pink), and unknown Al species (green).

It must be noted that ^29^Si ss-NMR does not detect aluminum not bonded through oxygen to silicon. The curve fits shown in Figure 2(a) give a framework Si:Al ratio of 30. Values between 24 and 37 were obtained when different curve-fitting programs and constraints were used. We note that there is a significant disagreement with the XRF results (Table 1). We will return to this point later.

^27^Al NMR of the zeolite was also carried out to provide an estimated percentage of the extra framework Al present in the ZSM-5 sample. Figure 2(b) shows the ^27^Al NMR of the fresh ZSM-5 sample (hydrated). The majority of the Al is present within the framework: tetrahedral Al (as AlO_4_) is seen as the major peak at 52.7 ppm (70% of the total intensity). The shoulder present at 33 ppm is usually found when the zeolite has undergone steaming. In steamed samples, this peak is dominant, whereas here it is more of a shoulder. About 7% of the NMR intensity is given by the peak present at −3 ppm, which is allocated to octahedral extra framework Al.^23^

The results of BET and pore volume measurements^24^ are shown in Table 2. (The data, Figure S1, and a detailed analysis are presented in the supporting information). The micropore volume is significantly smaller than most of those reported previously for ZSM-5 (typically 0.12–0.14 cm^3^ g^–1^),^25^ suggesting the possibility of extraneous material causing partial pore blockage.

**Table 2:** BET and Pore Volume Analysis of the ZSM-5

	total	micropore	mesopore	surface
BET/m^2^ g^–1^	370 ± 11	248 ± 9	72 ± 2	50 ± 2
volume/cm^3^ g^–1^		0.101 ± 0.003	0.032 ± 0.001	

### 3.2 Average Structure

The crystal structure of ZSM-5 has been investigated many times—the International Zeolite Association (IZA) database^26^ lists over 100 reports. The consensus is that ZSM-5 exhibits a “low-temperature” monoclinic form, space group **P**21̅*n*, and a “high-temperature” orthorhombic form, space group **Pnma**. For the end-member form silicalite, i.e., *n* = 0 in H_*n*_Al_*n*_Si_96–*n*_O_192_, the transition temperature is ∼340 K.^27^ This decreases with increasing Al content.^28,29^ However, the transition temperature is controversial: for zeolites similar to our sample, values of <272^28^ and ∼330 K^29^ have been proposed. The presence or otherwise of water is equally controversial: one report states that it has no effect on the transition temperature,^28^ another that it stabilizes the orthorhombic form.^29^

**Figure 3:**
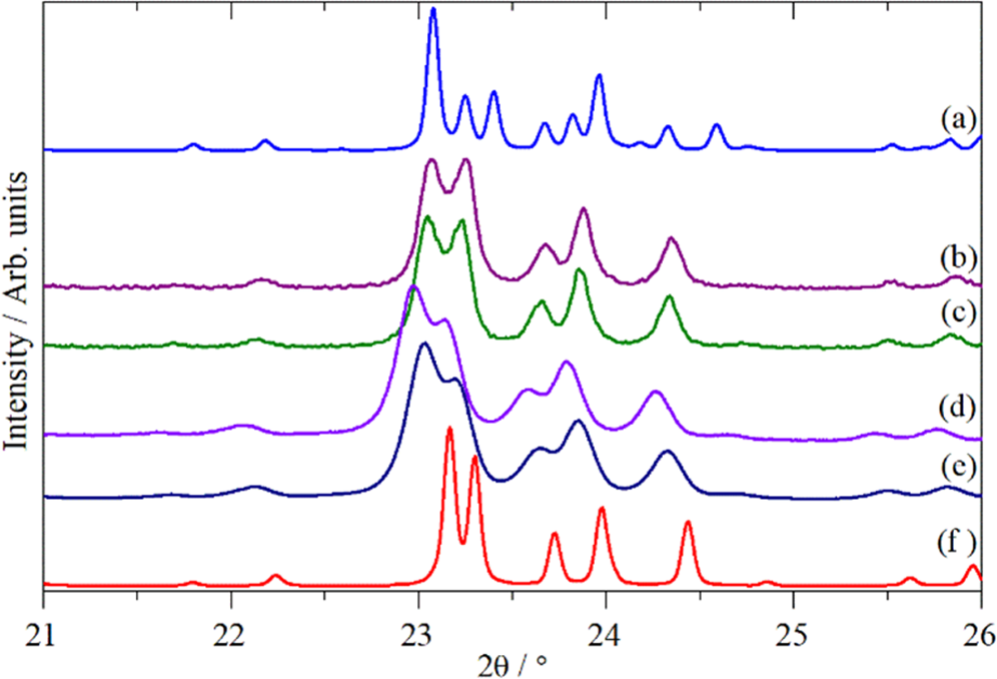
XRD patterns recorded for HZSM-5: (a) theoretical pattern calculated for monoclinic HZSM-5, (b) HZSM-5 at 13 K, (c) HZSM-5 at 300 K, (d) HZSM-% at room temperature after dehydration at 623 K, (e) same sample as (d) but after exposure to ambient air for 48 h, and (f) theoretical pattern calculated for orthorhombic HZSM-5 (all using λ = 1.5406 Å). The theoretical patterns were sourced from the IZA’s online structural database.^26^

Figure 3 compares powder XRD calculated from the literature^26^ for the monoclinic (3a) and orthorhombic forms (3f) with data recorded under various conditions. Figure 3b,c shows diffractograms recorded at 13 and 300 K, respectively (Figure S2 shows the entire temperature series between 13 and 300 K). Figure 3d shows a diffractogram from a sample that had been dehydrated at 623 K and loaded into a sealed can in a glovebox, and Figure 3e shows the same sample as Figure 3d after exposure to ambient air for 48 hours. Comparison of Figure 3f and Figure 3b–e suggests that all of the samples remain orthorhombic irrespective of the temperature and the presence or absence of water.

Powder X-ray diffraction analysis is capable of determining the overall arrangement of the zeolite framework: it is incapable of locating the hydrogens of the acid sites due to their negligible electron density. In an attempt to overcome the limitations of powder XRD, we have measured neutron powder diffraction (NPD) data from our material.

**Figure 4:**
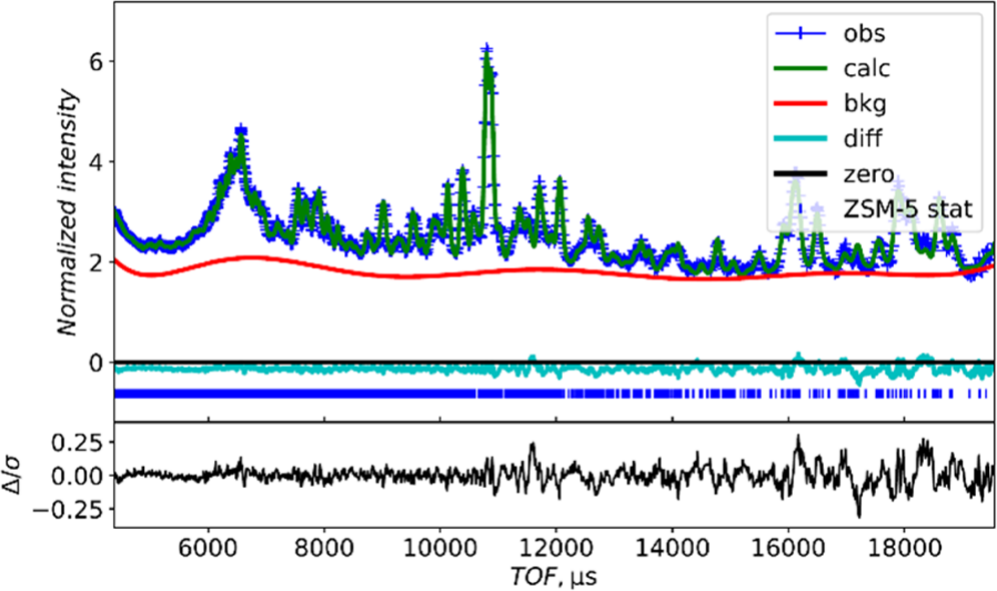
Fitted room-temperature Polaris bank 4 powder neutron diffraction pattern from HZSM-5 using the *P*21̅*n* monoclinic structure.

Crystal structure models were refined from these data in both the monoclinic and orthorhombic space groups. A comparison of the fits to the experimental data is shown in Figure 4 (monoclinic) and Figures S3 and S4 (orthorhombic). Both of these result in good agreement, with the monoclinic fit being marginally better. Table 3 lists the refined lattice parameters for the orthorhombic and monoclinic structure refinements. It can be seen that the monoclinic structure is very close to the orthorhombic one: the β angle deviates from 90° by less than 0.1°, and this is unobservable by conventional X-ray diffraction instrumentation. NPD is more sensitive to the oxygen positions than powder XRD and this probably accounts for why NPD finds the monoclinic structure and powder XRD finds the orthorhombic structure.

**Table 3:** Final Refined Unit Cell Parameters for Each Sample after Refinement in the Orthorhombic (o) Space Group **Pnma** and in the Monoclinic (m) Space Group *P*21̅*n*^a^

	*a (*Å)	*b* (Å)	*c* (Å)	β (°)	w*R* (B4)	w*R* (B5)
HZSM-5 (o)	19.9534(7)	20.1334(7)	13.4214(5)	90 (−)	1.82%	1.71%
HZSM-5 (m)	19.9534(7)	20.1334(7)	13.4214(5)	89.953(8)	1.22%	1.47%
silicalite	20.0511 (1)	19.8757 (1)	13.36823(9)	90 (−)		

a Silicalite^19^ is included for comparison.

Unfortunately, even though there is a modest difference between Si and Al neutron scattering lengths,^30^ it was not possible to differentiate between them in our refinements, presumably due to the small amount of Al present and the complexity of the structure, and it would be unwise to speculate further.

Artioli et al. studied silicalite by neutron diffraction^19^ and found evidence for vacancies on some of the silicon sites of the orthorhombic model, but not in the monoclinic model. This was tested in the current work, but there was no convincing evidence for any significant vacancies, as in Artioli’s monoclinic model. Additionally, due to the (necessary) constraints imposed upon the displacement parameters (all Si the same), the correlation between U and site occupancy could potentially lead to misinterpretation.

From the initial model, a Fourier difference map was generated. This approach did not produce any candidates for intercalated atoms or for the location of H/D. Charge flipping^31^ was then used, and here, it was possible to identify extra-framework atoms at a reasonable distance from the framework (∼2.5 Å). This could be interpreted on the basis of the extra-framework Al proposed by Holzinger et al. from solid-state ^27^Al NMR data^32^ and by Ravi et al.,^33^ but further work would be required with higher resolution data to test this hypothesis. At present, therefore, the structural analysis is incomplete but does provide a good basis for further work.

Tables S1 and S2 list the refined atomic coordinates for the orthorhombic and monoclinic models.

### 3.3 Acid Sites

**Figure 5:**
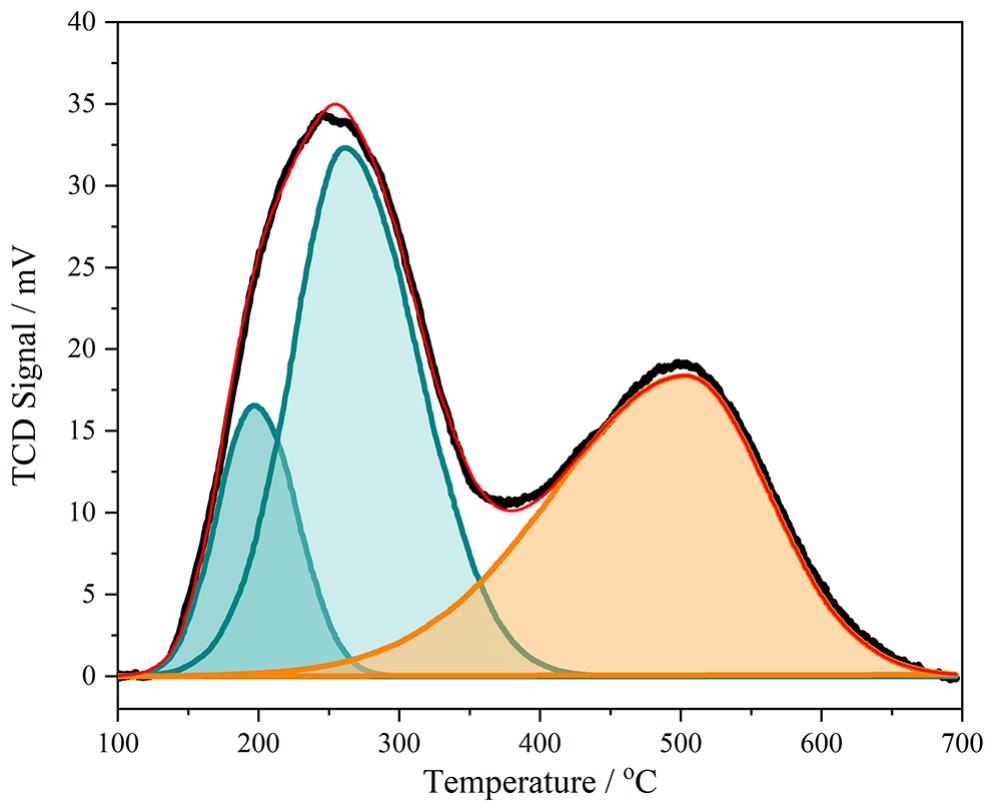
Ammonia TPD of calcined HZSM-5, together with peak fitting of the experimental data. The experimental data (black) was background-subtracted, owing to the TCD signal being nonzero at higher temperatures. The total fit to the experimental data (red), the two peaks needed to fit the low-temperature peak (green), and one peak needed to fit the high-temperature peak (orange) are shown.

Ammonia TPD is a commonly used method for the quantitative determination of the number of acid sites present.^20^Figure 5 shows the result for our material. Three Gaussian functions are needed to fit the desorption peaks. The low-temperature peaks are assigned to chemisorption at silanol sites, potentially with additional contributions from adsorption on extra-framework species or residual physisorbed ammonia. The high-temperature peak, with a peak maximum at 530 °C is associated with Brønsted acid sites.^34^ From peak fitting the desorption peaks, the high-temperature peak associated with Brønsted acidity showed that there were 2.43 Brønsted sites/HZSM-5 unit cell, which corresponds to a framework Si:Al of 38.5, assuming one acid site for every aluminum in the lattice.

**Figure 6:**
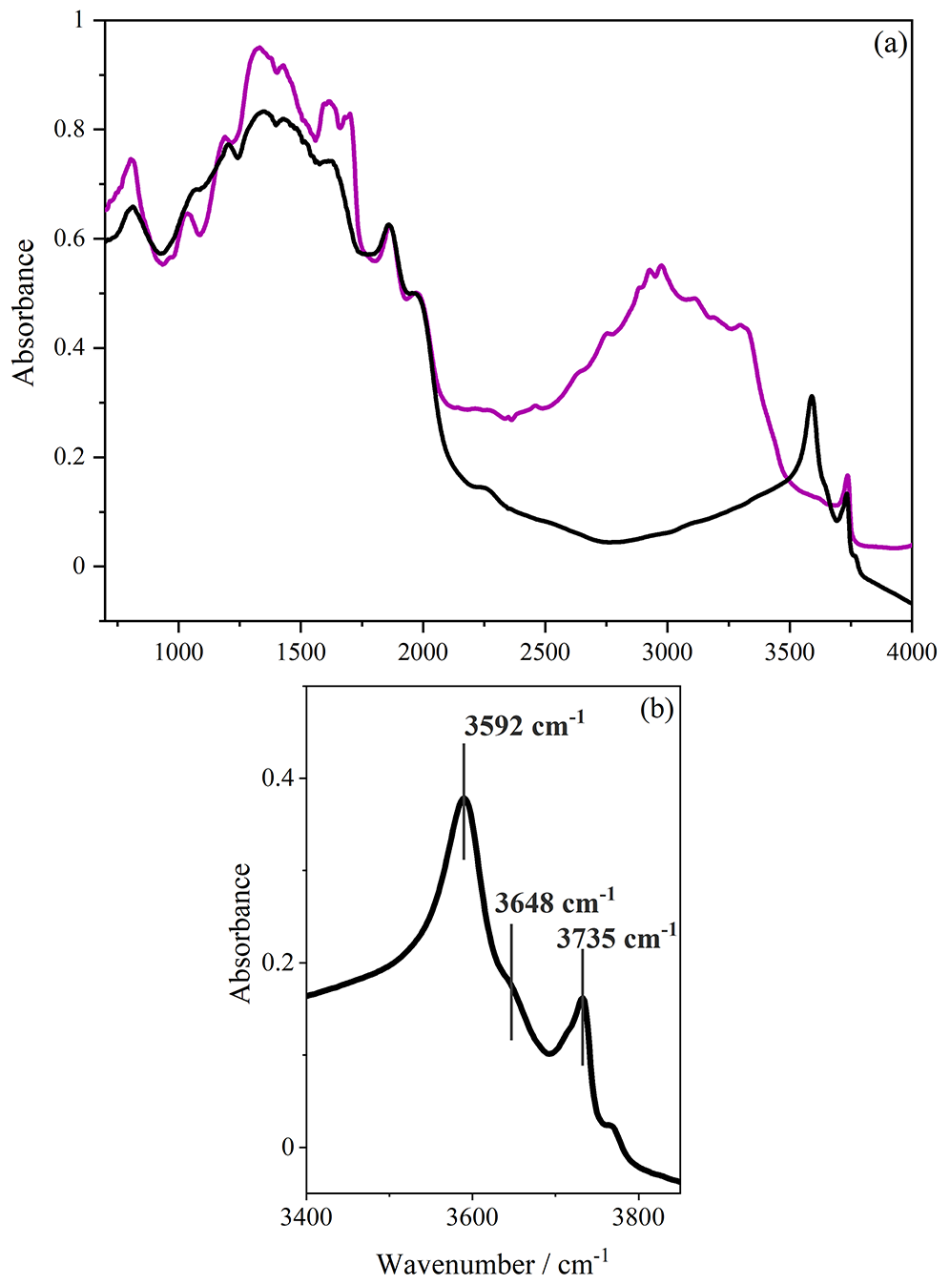
DRIFTS spectra of ZSM-5 (a) as received (purple) and after it has been calcined and dried (black). (b) Detailed view of the acid sites of calcined and dried ZSM-5 spectrum. Both spectra were measured at 350 °C under a nitrogen atmosphere.

Figure 6 shows the infrared spectrum of our ZSM-5, as received after drying at 350 °C (Figure 6a, purple trace) and after calcination and drying (Figure 6a,b black trace). In Figure 6a, the template is clearly visible as broad peaks in the 3200–2500 cm^–1^ region. Upon calcination, the template is seen to have disappeared and the spectrum of ZSM-5 resembles that seen in the literature.^35^ Below 2000 cm^–1^, the spectrum is dominated by the Si–O and Al–O framework stretch modes and their overtones. These are not associated directly with the active sites. However, the spectrum does provide useful information on the O–H stretch modes located above 3500 cm^–1^.^11^ The strongest of these is at 3592 cm^–1^ and is assigned to Brønsted acid groups within the zeolite. The weaker, separate peak at 3735 cm^–1^ results from the silanol Si–O–H groups that terminate the zeolite framework at its exterior surfaces and at internal defects. The peak between these two modes at 3648 cm^–1^ is associated with the presence of extra-framework aluminum species.

**Figure 7:**
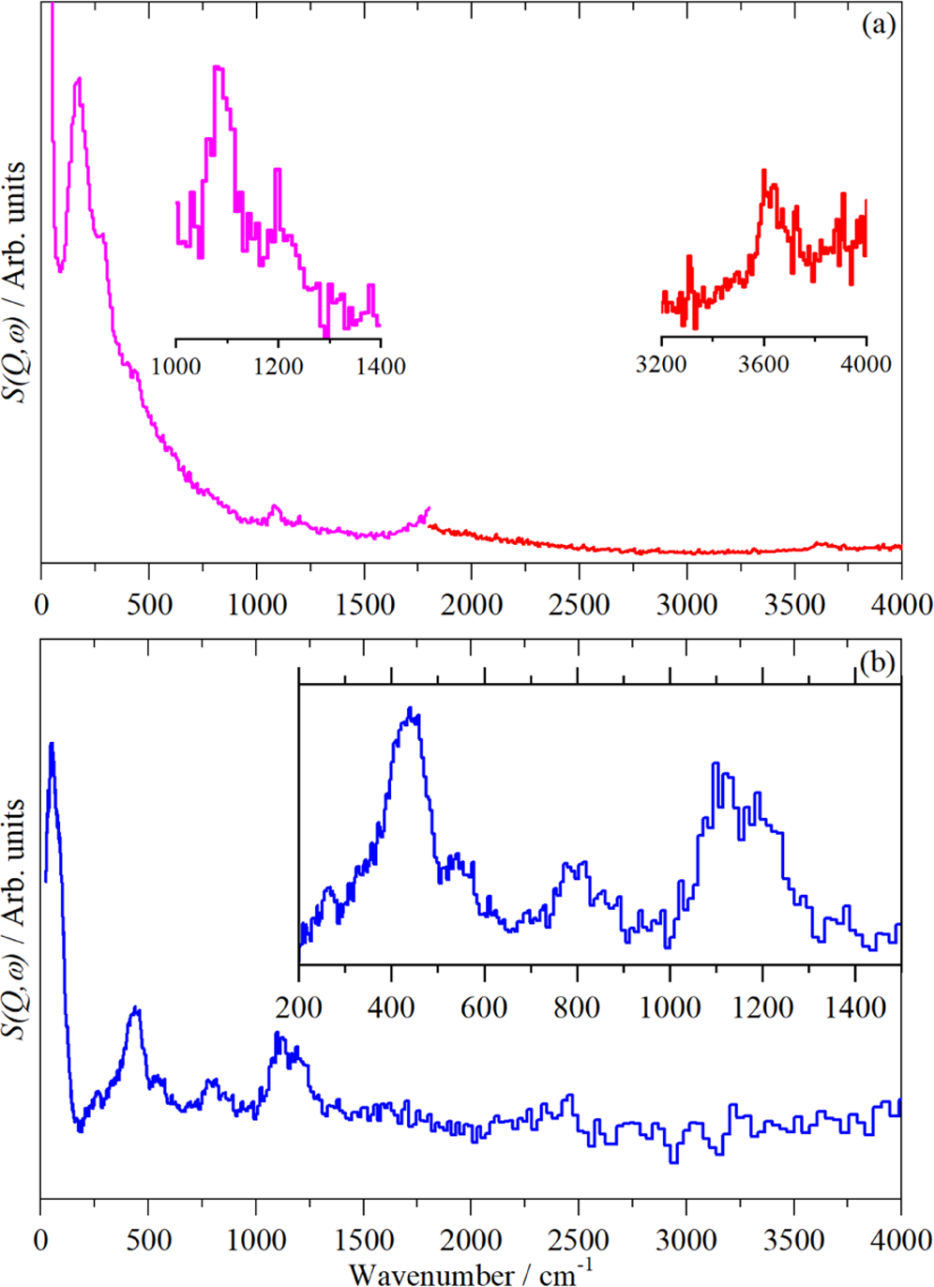
INS spectra of calcined, dried ZSM-5 at T ≤ 30 K. (a) Recorded with MAPS using incident energies of 2017 cm^–1^ (left) and 5244 cm^–1^ (right) and integrated over the momentum transfer range 0 ≤ *Q* ≤ 10 Å^–1^. Insets show × 10 expansions of selected regions to make the O–H-related modes more apparent. (b) Recorded with TOSCA. The insets show × 4 ordinate expansion of the internal mode region.

The corresponding spectra as measured by INS are shown in Figure 7a,b for data recorded on MAPS and TOSCA, respectively. The low intensity of the zeolite spectra owing to the small scattering cross sections of the framework atoms is immediately apparent. The complementarity of the instruments is evident. The reasons for this are explained elsewhere.^14^

While the scattering cross sections of Al, Si, and O are only ∼10% that of ^1^H (∼80 barn),^30^ there are more than 10 times as many of them as there are hydrogen atoms. Hence, they would be expected to appear in the spectra. A comparison with the INS spectra of a variety of silicas^36,37^ shows that the modes at 445, 535, 800, 1115, and 1205 cm^–1^ are all due to Si–O modes, the two high-energy modes are stretches and the remainder are deformations. The O–H stretching modes are observed by MAPS.

The intensity of a mode in the INS depends on the momentum transfer (*Q*, Å^–1^) and the amplitude of vibration of the atoms in the mode and their scattering cross section.^14^ In the harmonic approximation, the amplitude of vibration depends only on the reduced mass of the atoms and the energy transfer. This means that the intensity per oscillator is independent of the material, i.e., the “extinction coefficient” of the C–H stretch of an alkane is the same for all sp^3^ C–H modes. It does not depend on the electronic structure of the molecule as is the case for infrared and Raman spectroscopies. Hence, by calibration with a suitable reference material (one that has modes close to the mode of interest), it is possible to quantify the number of oscillators in the beam. This procedure has been used to quantify the amount of OH and CH in the hydrocarbonaceous overlayers on methane reforming^38^ and Fischer–Tropsch catalysts.^39^

**Figure 8:**
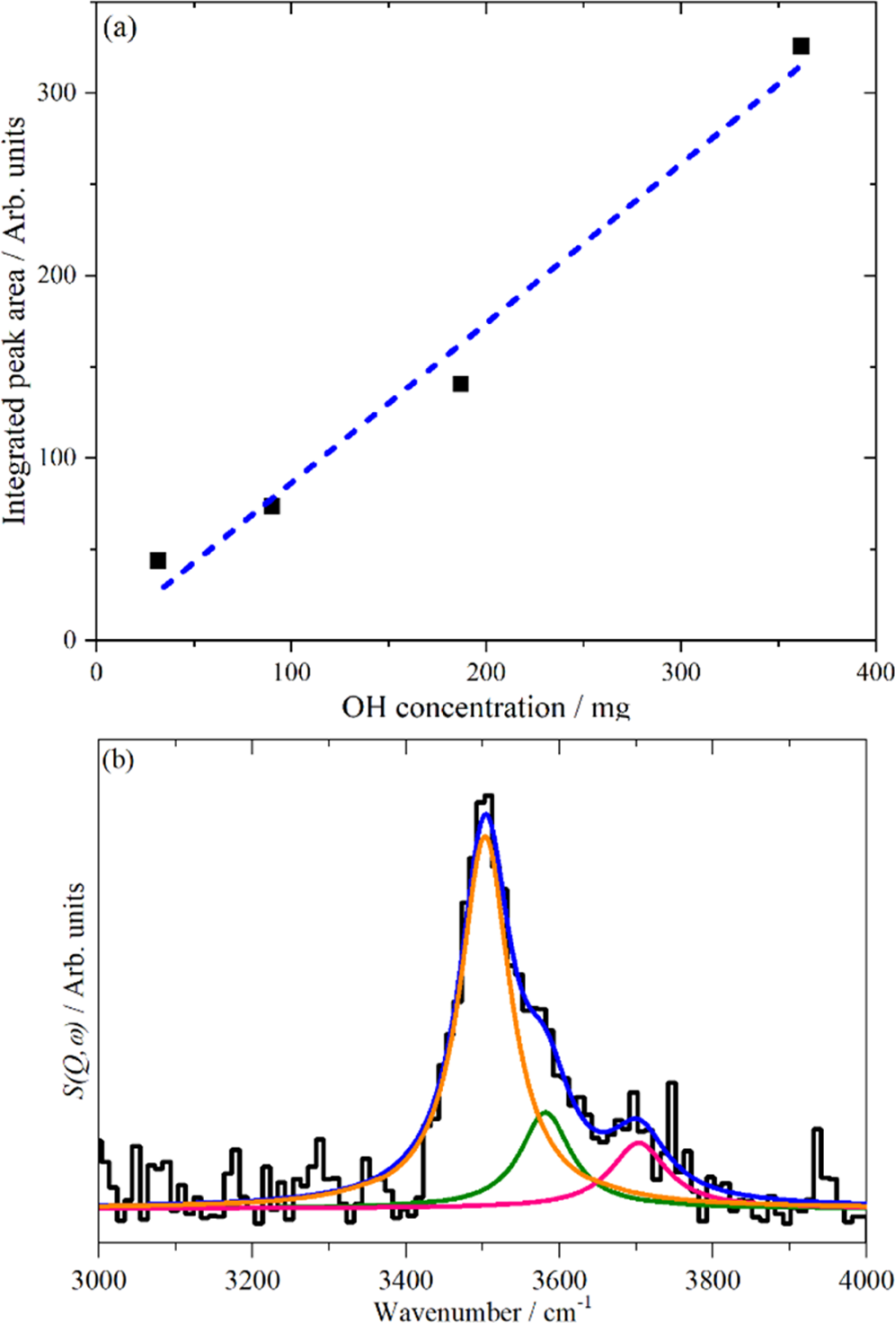
(a) INS OH calibration generated using the integrated areas of the O–H stretch of brucite peaks at different concentrations. (b) INS spectrum of ZSM-5 in the O–H stretch region. Key: black, ZSM-5 spectrum; orange, fitted curve for the Brønsted peak; green, extra framework aluminum; pink, silanols; and blue, total fit.

The INS spectrometer was calibrated by measuring a series of brucite, Mg(OH)_2_, samples of different weights (Figure 8a). This calibration was then applied to the ZSM-5 sample. The INS spectrum in the O–H stretch region was resolved into the three components identified above, and the amounts of each type of hydroxyl group were determined (Figure 8b). The INS quantification gives 3.26 OH Brønsted acid sites in an HZSM-5 unit cell (Table 4). Equating the Brønsted acid site concentration to aluminum in the zeolite framework gives a framework Si:Al ratio of 28.

**Table 4:** Quantification of the OH Sites of the Fresh HZSM-5

OH site	OH concentration mg (*g*_ZSM-5_)^−1^	OH/unit cell
Brønsted	9.77	3.26
extra-framework Al	3.32	1.11
silanol	1.98	0.66

**Figure 9:**
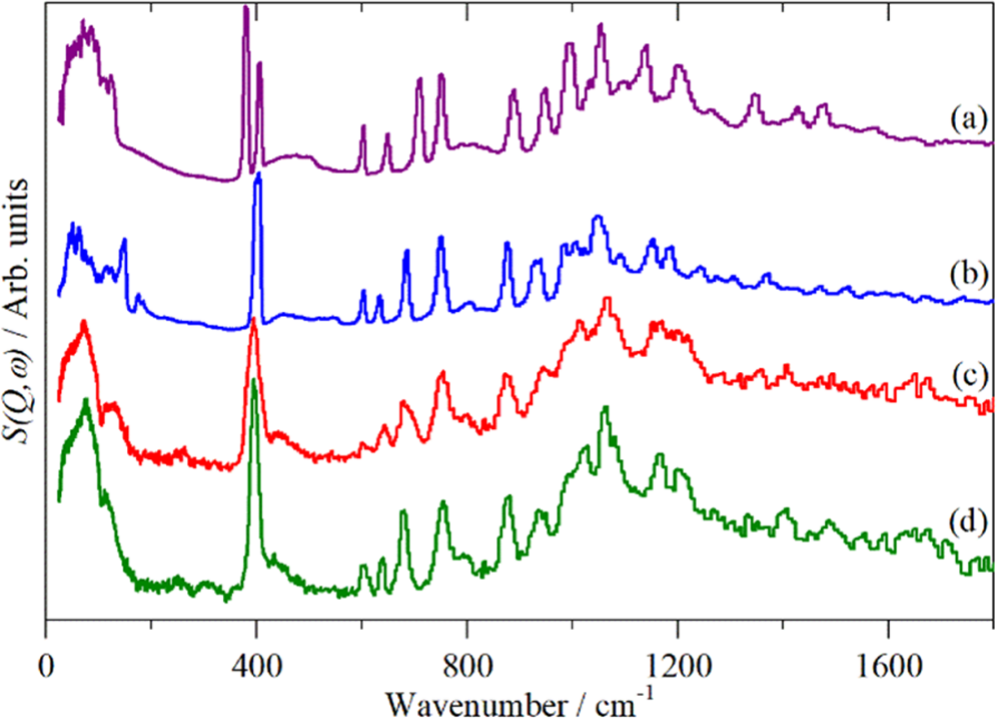
TOSCA INS spectra: (a) pyridine, (b) pyridinium chloride, (c) after pyridine adsorption on dried HZSM-5 at 373 K, and (d) after desorption from HZSM-5 (sample shown in (c)) at 523 K.

A zeolite may have both Lewis and Brønsted acidities; the former arises from coordinatively unsaturated aluminum ions.^33^ Infrared spectroscopy of chemisorbed pyridine has long been used to characterize Lewis and Brønsted acidity on materials.^40^ An experiment where pyridine was used as a probe molecule to study the strength of the acid sites was designed using INS as it should allow quantification of the number and type of acid site. Figure 9a,b shows the INS spectra of the reference compounds pyridine and pyridinium chloride. Understanding the differences between the spectra is important to identify if the pyridine in the zeolites is in its molecular form (i.e., on a Lewis acid site) or if it is chemisorbed in the form of the pyridinium ion (i.e., it has reacted with a Brønsted site). The most obvious difference in the spectra is that pyridine has a doublet at 378 and 406 cm^–1^, whereas pyridinium chloride only has one peak at 404 cm^–1^. Another distinct difference is that pyridine has a sharp peak at 991 cm^–1^. This peak is present in the pyridinium chloride only as a shoulder to the 1047 cm^–1^ peak rather than as a distinct peak. Pyridine also has sharp peaks at 1351, 1427, and 1478 cm^–1^, whereas the peaks are not as sharp or obvious in the pyridinium chloride spectrum.

Figure 9c,d shows the fresh HZSM-5, after the pyridine adsorption, and after desorption at 523 K, as measured on TOSCA. Both spectra resemble more closely the pyridinium chloride spectrum (Figure 9b). The initial adsorption spectrum shows broadened peaks, which suggests that an excess of pyridine is also present. The peaks become sharper after desorption, with the only pyridine present within the zeolite being in its pyridinium ion form, chemisorbed on the zeolite hydroxyls, as shown by the single peak at 400 cm^–1^.

**Figure 10:**
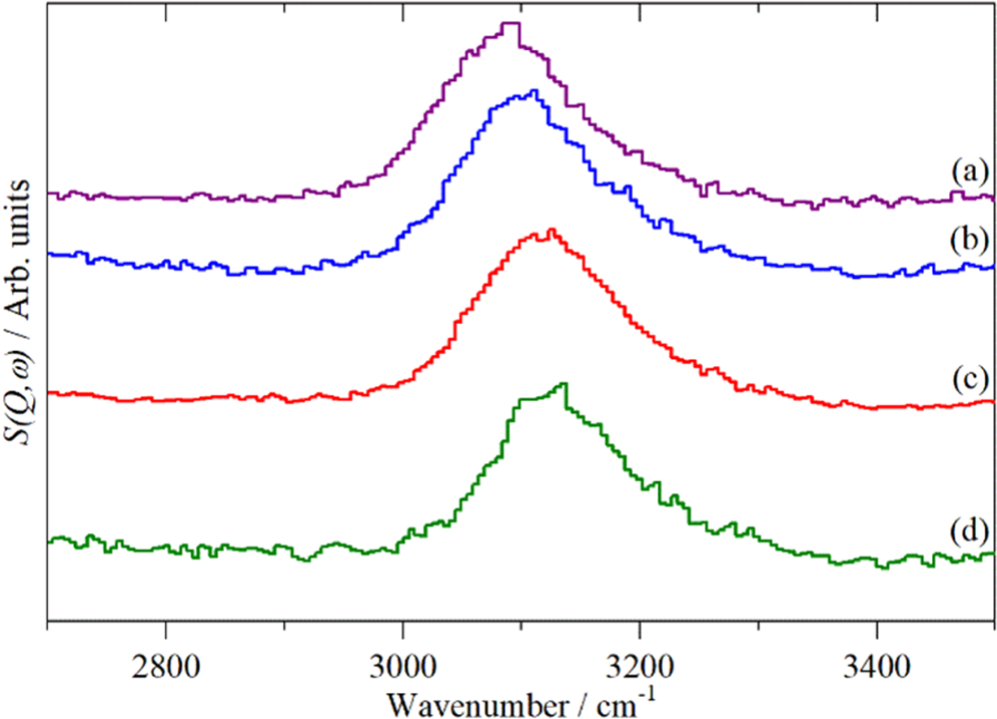
MAPS INS spectra: (a) pyridine, (b) pyridinium chloride, (c) after pyridine adsorption on dried ZSM-5 at 373 K, and (d) after desorption from ZSM-5 (sample shown in (c)) at 523 K. (d) ×4 Ordinate expanded relative to (c).

Figure 10 shows the corresponding MAPS spectra. The samples are not the same as those shown in Figure 9, but they were prepared in the same way. Figure 10a,b shows the reference compounds. The pyridine peak maximum is at 3086 cm^–1^, whereas in pyridinium chloride, the peak is shifted slightly to a peak maximum of 3106 cm^–1^. This small shift will be used to identify which form of pyridine is present on the zeolite. It is seen that there is a significant drop in the intensity after the pyridine desorption step (note the different ordinate scales in (b) and (c)). The peak maximum is at 3120 cm^–1^, which shifts to 3135 cm^–1^ after pyridine desorption. This means that the peak is a mixture of pyridine and pyridinium. Together with the significant weight loss, this shows that even with the adsorption being carried out at 373 K and flushing the sample after dosing, there is some physisorbed pyridine present. Unfortunately, this masks any pyridine adsorbed on Lewis acid sites. All of the peak maxima are seen to be a little higher than the pyridinium chloride (both before and after pyridine desorption). This shift in the peak maximum is probably because the pyridinium ion in the zeolite is in a different environment to that present in the chloride salt.

The amount of pyridinium calculated from the INS peak in the desorbed sample gives a Si:Al ratio of 36:1. From the mass change of the desorbed sample and assuming a 1:1 ratio of Al:pyridine, we obtain a Si:Al ratio of 26:1, which is close to the ratio obtained from the direct quantification of the hydroxyls by INS.

## 4 Discussion

As stated in the Section 1, knowledge of the Si:Al ratio is a key piece of catalytically relevant information. Table 5 collates the results from the various techniques. It can be seen from the variation in Table 5 that the Si:Al ratio is an elusive number and depends very much on how it is determined. We believe that the differences arise because the techniques are not measuring the same parameters. XRF measures the entire sample, so gives a ratio that includes all of the aluminum, whether or not it is present in the framework. The ^29^Si ss-NMR and the INS hydroxyl methods are both specific for framework Brønsted sites, so give very similar results. Ammonia TPD and the INS pyridine method both rely on protonation of the probe molecule, so their higher values are possibly an indication that there are acid sites with different strengths. The INS pyridine method also assumes that all of the sample is in the neutron beam; this may account for the difference in the ratio determined from the mass change. For catalytic use, the most relevant values are those that only include the aluminum that is present in a Brønsted site; hence, we conclude that our material has a Si:Al ratio of 30±5:1, as previously stated.^41^

**Table 5:** Summary of the Si:Al Ratio in ZSM-5 Determined by Different Methods

method	Si:Al
XRF (analysis 1)	16.6:1
XRF (analysis 2)	16.3:1
^29^Si ss-NMR	30:1
ammonia TPD	38.5:1
INS (hydroxyls)	28:1
INS (pyridine)	36.5:1
pyridine gravimetry	26:1

The difference between the XRF value of ∼16:1 and the “true” value of 30:1 shows that almost half of the aluminum is not in the zeolite lattice. This is generically known as extra framework aluminum (EFAl), and its nature has been the subject of extensive work, e.g., refs (11, 33, 42, 43). The ^27^Al-ssNMR spectrum of our material (Figure 2b) shows that ∼30% of the Al is present as EFAl of some sort. Al^3+^ in non-hydrogen-bonded environments is invisible to conventional NMR because of the large ^27^Al quadrupole coupling constants,^11^ and this probably accounts for the difference between the XRF and ^27^Al ss-NMR results. Our INS results (Table 4) show that only one-third or so of the EFAl aluminum ions have hydroxyls associated with them. Several of the postulated EFAl species have one or more hydroxyl groups attached;^42^ this work suggests that these are actually minority species.

The presence of the EFAl also helps to explain why the analysis of the neutron diffraction data was so problematic. The EFAl is proposed to consist of small clusters that are randomly distributed throughout the structure. Thus, it would contribute diffuse scattering that appears as broad features in the background, rather than in the Bragg reflections. This is in agreement with both the H-ZSM-5 and the D-ZSM-5 samples having comparable background levels; usually, the deuterated sample would be expected to have a significantly lower background because of the reduced incoherent scattering of deuterium. A significant quantity of EFAl would also explain the unusually low pore volume of this material.

## 5 Conclusions

In this work, we have comprehensively characterized an industrial ZSM-5 catalyst. We have shown that the Si:Al ratio that is determined depends very much on the method that is used for its measurement, and we explain how these differences arise.

In addition to conventional techniques, we have also exploited neutron scattering methods to provide new information. In principle, neutron diffraction is capable of determining the positions of all of the atoms present, including the crucial Al and H atoms. In practice, this has proven to be impossible in this case. The reasons why are instructive. In this commercial material, ∼50% of the Al atoms are not in the zeolite lattice. This has two consequences; it makes for a strong diffuse scattering background and it also reduces the number of framework Al atoms, reducing their already small contribution to the Bragg reflection intensities. It is also possible that there is no preferred site for the Al (^27^Al NMR studies suggest that multiple sites are occupied^32,33^), which would mean that the Al and hence the H are randomly distributed throughout the lattice and the peak intensities would depend on the weighted average of the Si and Al scattering lengths. The presence of the diffuse scattering is consistent with (but does not prove) the framework Al being much less random and the short-range ordering results in diffuse scattering. Single-crystal neutron diffraction would overcome many of these problems; unfortunately, crystals of suitable size are not available.

INS spectroscopy provides complementary information to that obtainable by infrared spectroscopy. The ability to both quantify and speciate the hydroxyls is unique and has clear potential for other microporous systems. The ability of INS to provide access to the entire “mid-infrared” means that all of the modes of adsorbed pyridine can be used, rather than just the two or three that are used for infrared studies. We have shown elsewhere^44^ that the low-energy modes of pyridine undergo much larger shifts than the ring modes used in infrared spectroscopy, potentially allowing greater discrimination between acid site strengths. In this work, the spectra are dominated by pyridinium ions, and the presence of residual physisorbed pyridine meant that we could not observe the Lewis acid sites. An improved experimental protocol could remove this limitation.

## Supporting Information Available

Surface area and volume measurements, variable-temperature powder XRD data, comparison of observed and calculated NPD patterns based on the monoclinic structure, and refined positions for the orthorhombic model (PDF)

## References

[ref1] Chester A. W.; Derouane E. G. Zeolite Characterisation and Catalysis; Springer: Dordrecht, 2009 10.1007/978-1-4020-9678-5.

[ref2] Weitkamp J. Zeolites and catalysis. Solid State Ionics 2000, 131, 175–188. 10.1016/S0167-2738(00)00632-9.

[ref3] Argauer R. J.; Landolt G. R. Crystalline Zeolite zsm-5 and Method of Preparing the Same. US3,702,886,1972.

[ref4] Degnan T. F.; Chitnis G. K.; Schipper P. H. History of ZSM-5 fluid catalytic cracking additive development at Mobil. Microporous Mesoporous Mater. 2000, 35-36, 245–252. 10.1016/S1387-1811(99)00225-5.

[ref5] den Hollander M. A.; Wissink M.; Makkee M.; Moulijn J. A. Gasoline conversion: reactivity towards cracking with equilibrated FCC and ZSM-5 catalysts. Appl. Catal., A 2002, 223, 85–102. 10.1016/S0926-860X(01)00745-1.

[ref6] Olson D. H.; Haag W. O. Structure-Selectivity Relationship in Xylene Isomerization and Selective Toluene Disproportionation. In Catalytic Materials: Relationship Between Structure and Reactivity; Whyte T. E.; Dalla Betta R. A.; Derouane E. G.; Baker R. T. K., Eds.; ACS Symposium Series, 1984; Chapter. 14, pp 275–307 10.1021/bk-1984-0248.ch014.

[ref7] Yarulina I.; Chowdhury A. D.; Meirer F.; Weckhuysen B. M.; Gascon J., Recent trends and fundamental insights in the methanol-to-hydrocarbons process. Nat. Catal. 2018 1 398, 411, https://dx.doi.org/10.1038/s41929-018-0078-5, 10.1038/s41929-018-0078-5.

[ref8] Awayssa O.; Al-Yassir N.; Aitani A.; Al-Khattaf S. Modified HZSM-5 as FCC additive for enhancing light olefins yield from catalytic cracking of VGO. Appl. Catal., A 2014, 477, 172–183. 10.1016/j.apcata.2014.03.021.

[ref9] Olsbye U.; Svelle S.; Lillerud K. P.; Wei Z. H.; Chen Y. Y.; Li J. F.; Wang J. G.; Fan W. B. The formation and degradation of active species during methanol conversion over protonated zeotype catalysts. Chem. Soc. Rev. 2015, 44, 7155–7176. 10.1039/c5cs00304k. 26185806

[ref10] Warringham R.; Bellaire D.; Parker S. F.; Taylor J.; Ewings R. A.; Goodway C. M.; Kibble M.; Wakefield S. R.; Jura M.; Dudman M. P.; Tooze R. P.; Webb P. B.; Lennon D. Sample Environment Issues Relevant to the Acquisition of Inelastic Neutron Scattering Measurements of Heterogeneous Catalyst Samples. In Journal of Physics: Conference Series; IOP Publishing, 2014; Vol. 554 012005 10.1088/1742-6596/554/1/012005.

[ref11] Campbell S. M.; Bibby D. M.; Coddington J. M.; Howe R. F.; Meinhold R. H. Dealumination of HZSM-5 zeolites: I. Calcination and hydrothermal treatment. J. Catal. 1996, 161, 338–349. 10.1006/jcat.1996.0191.

[ref12] de Boer J. H.; Lippens B. C.; Linsen B. G.; Broekhoff J.C.P.; van den Heuvel A.; Osinga T. J. The *t*-curve of multimolecular N_2_-adsorption. J. Colloid Interface Sci. 1966, 21, 405–414. 10.1016/0095-8522(66)90006-7.

[ref13] Pinna R. S.; Rudić S.; Parker S. F.; Armstrong J.; Zanetti M.; Škoro G.; Waller S. P.; Zacek D.; Smith C. A.; Capstick M. J.; McPhail D. J.; Pooley D. E.; Howells G. D.; Gorini G.; Fernandez-Alonso F. The neutron guide upgrade of the TOSCA spectrometer. Nucl. Instrum. Methods Phys. Res. A 2018, 896, 68–74. 10.1016/j.nima.2018.04.009.

[ref14] Parker S. F.; Lennon D.; Albers P. W. Vibrational spectroscopy with neutrons – a review of new directions. Appl. Spectrosc. 2011, 65, 1325–1341. 10.1366/11-06456.

[ref15] Ewings R. A.; Stewart J. R.; Perring T. G.; Bewley R. I.; Le M. D.; Raspino D.; Pooley D. E.; Škoro G.; Waller S. P.; Zacek D.; Smith C. A.; Riehl-Shaw R. C. Upgrade to the MAPS neutron time-of-flight chopper spectrometer. Rev. Sci. Inst. 2019, 90, 035110 10.1063/1.5086255. 30927771

[ref16] Smith R. I.; Hull S.; Tucker M. G.; Playford H. Y.; McPhail D. J.; Waller S. P.; Norberg S. T. The upgraded Polaris powder diffractometer at the ISIS neutron source. Rev. Sci. Instrum. 2019, 90, 115101 10.1063/1.5099568. 31779457

[ref17] Toby B. H.; Dreele R. B. Von. *GSAS-II*: the genesis of a modern open-source all purpose crystallography software package. J. Appl. Crystallogr. 2013, 46, 544–549. 10.1107/S0021889813003531.

[ref18] van Koningsveld H.; Jansen J. C.; van Bekkum H. The monoclinic framework structure of zeolite H-ZSM-5. Comparison with the orthorhombic framework of as-synthesized ZSM-5. Zeolites 1990, 10, 235–242. 10.1016/0144-2449(94)90134-1.

[ref19] Artioli G.; Lamberti C.; Marra G. L. Neutron powder diffraction study of orthorhombic and monoclinic defective silicalite. Acta Crystallogr., Sect. B: Struct. Sci. 2000, 56, 2–10. 10.1107/S0108768199008927. 10735438

[ref20] Arena F.; Di Chio R.; Trunfio G. An experimental assessment of the ammonia temperature programmed desorption method for probing the surface acidic properties of heterogeneous catalysts. Appl. Catal., A 2015, 503, 227–236. 10.1016/j.apcata.2015.05.035.

[ref21] Fyfe C. A.; Feng Y.; Grondey H.; Kokotailo G. T.; Gies H. One- and two-dimensional high-resolution solid-state NMR studies of zeolite lattice structures. Chem. Rev. 1991, 91, 1525–1543. 10.1021/cr00007a013.

[ref22] Lowenstein W. The distribution of aluminum in the tetrahedra of silicates and aluminates. Am. Mineral.: J. Earth Planet. Mater. 1954, 39, 92–96.

[ref23] Triantafillidis C. S.; Vlessidis A. G.; Nalbandian L.; Evmiridis N. P. Effect of the degree and type of the dealumination method on the structural, compositional and acidic characteristics of H-ZSM-5 zeolites. Microporous Mesoporous Mater. 2001, 47, 369–388. 10.1016/S1387-1811(01)00399-7.

[ref24] Sing K. S. W. Reporting physisorption data for gas/solid systems with special reference to the determination of surface area and porosity (Recommendations 1984). Pure Appl. Chem. 1985, 57, 603–619. 10.1351/pac198557040603.

[ref25] Remy M. J.; Poncelet G. A new approach to the determination of the external surface and micropore volume of zeolites from the nitrogen adsorption isotherm at 77 K. J. Phys. Chem. A 1995, 99, 773–779. 10.1021/j100002a047.

[ref26] Baerlocher C.; McCusker L. Database of Zeolite Structures. http://www.iza-structure.org/databases/. (accessed Aug 2021).

[ref27] van Koningsveld H.; Jansen J. C.; van Bekkum H. The orthorhombic/monoclinic transition in single crystals of zeolite ZSM-5. Zeolites 1987, 7, 564–568. 10.1016/0144-2449(87)90099-6.

[ref28] Mentzen B. F.; Letoffe J.-M.; Claudy P. Enthalpy change and temperature of the reversible monoclinic-orthorhombic phase transition in MFI type zeolitic materials. Thermochim. Acta 1996, 288, 1–7. 10.1016/0040-6031(96)89216-1.

[ref29] Hay D. G.; Jaeger H. Orthorhombic-monoclinic phase changes in ZSM-5 zeolite/silicalite. J. Chem. Soc., Chem. Commun. 1984, 1433 10.1039/C39840001433.

[ref30] Sears V. F. Neutron scattering lengths and cross sections. Neutron News 1992, 3, 26–37. 10.1080/10448639208218770.

[ref31] Oszlányi G.; Süto A. *Ab initio* structure solution by charge flipping. Acta Crystallogr., Sect. A: Found. Crystallogr. 2004, 60, 134–141. 10.1107/S0108767303027569. 14966324

[ref32] Holzinger J.; Beato P.; Lundegaard L. F.; Skibsted J. Distribution of aluminum over the tetrahedral sites in ZSM-5 Zeolites and their evolution after steam treatment. J. Phys. Chem. C 2018, 122, 15595–15613. 10.1021/acs.jpcc.8b05277.

[ref33] Ravi M.; Sushkevich V. L.; van Bokhoven J. A. Towards a better understanding of Lewis acidic aluminium in zeolites. Nat. Mater. 2020, 19, 1047–1056. 10.1038/s41563-020-0751-3. 32958864

[ref34] Lónyi F.; Valyon J. On the interpretation of the NH_3_-TPD patterns of H-ZSM-5 and H-mordenite. Microporous Mesoporous Mater. 2001, 47, 293–301. 10.1016/S1387-1811(01)00389-4.

[ref35] Zecchina A.; Spoto G.; Bordiga S. Probing the acid sites in confined spaces of microporous materials by vibrational spectroscopy. Phys. Chem. Chem. Phys. 2005, 7, 1627–1642. 10.1039/B418763F. 19787918

[ref36] Albers P. W.; Michael G.; Rotgerink H. L.; Parker S. F. Low frequency vibrational dynamics of amorphous and crystalline silica. J. Z. Naturforsch., B 2012, 67, 1016–1020. 10.5560/ZNB.2012-0110.

[ref37] Parker S. F.; Klehm U.; Albers P. W. Differences in the morphology and vibrational dynamics of crystalline, glassy and amorphous silica – commercial implications. Mater. Adv. 2020, 1, 749–759. 10.1039/D0MA00158A.

[ref38] Silverwood I. P.; Hamilton N. G.; Laycock C. J.; Staniforth J. Z.; Ormerod R. M.; Frost C. D.; Parker S. F.; Lennon D. Quantification of surface species present on a nickel/alumina methane reforming catalyst. Phys. Chem. Chem. Phys. 2010, 12, 3102–3107. 10.1039/B919977B. 20237696

[ref39] Davidson A. L.; Gibson E. K.; Cibin G.; Vanrensburg H.; Parker S. F.; Webb P. B.; Lennon D. The application of inelastic neutron scattering to investigate iron-based Fischer-Tropsch to olefins catalysis. J. Catal. 2020, 392, 197–208. 10.1016/j.jcat.2020.09.025.

[ref40] Kung M. C.; Kung H. H. IR studies of NH_3_, pyridine, CO, and NO adsorbed on transition metal oxides. Catal. Rev. 1985, 27, 425–460. 10.1080/01614948508064741.

[ref41] Howe R. F.; Gibson E. K.; Catlow C. R.; Hameed A.; McGregor J.; Collier P.; Parker S. F.; Lennon D. An assessment of hydrocarbon species in the methanol-to-hydrocarbon reaction over a ZSM-5 catalyst. Faraday Disc. 2017, 197, 447–471. 10.1039/c6fd00195e. 28194458

[ref42] Yi X.; Liu K.; Chen W.; Li J.; Xu S.; Li C.; Xiao Y.; Liu H.; Guo X.; Liu S.-B.; Zheng A. Origin and structural characteristics of tri-coordinated extra-framework aluminum species in dealuminated zeolites. J. Am. Chem. Soc. 2018, 140, 10764–10774. 10.1021/jacs.8b04819. 30070481

[ref43] Sklenak S.; Dědeček J.; Li C.; Wichterlová B.; Gábová V.; Sierka M.; Sauer J. Aluminium siting in the ZSM-5 framework by combination of high resolution ^27^Al NMR and DFT/MM calculations. Phys. Chem. Chem. Phys. 2009, 11, 1237–1247. 10.1039/B807755J. 19209368

[ref44] Lundie D. T.; McInroy A. R.; Marshall R.; Winfield J. M.; Mitchell C.; Dudman C. C.; Jones P.; Parker S. F.; Lennon D. An improved description of the surface acidity of eta-alumina. J. Phys. Chem. B 2005, 109, 11592–11601. 10.1021/jp0405963. 16852423

[ref45] Lennon D.; Zachariou A.; Hawkins A. P.; Collier P.; Parker S. F. Studying the Strength of Acid Sites in an Industrial ZSM5 Catalyst: MAPS STFC ISIS Neutron Muon Source 2018 10.5286/ISIS.E.RB1910561.

[ref46] Lennon D.; Zachariou A.; Hawkins A. P.; Collier P.; Parker S. F. POLARIS Xpress STFC ISIS Neutron Muon Source 2019 10.5286/ISIS.E.RB1990175-1.

