# Supplementary material for: Counting the Acid Sites in a Commercial ZSM-5 Zeolite Catalyst
Source: ACS Phys Chem Au. 2022 Nov 1;3(1):74–83. doi: 10.1021/acsphyschemau.2c00040 (PMC9881239; doi:10.1021/acsphyschemau.2c00040)
Supplement: Supplementary file 1 — pg2c00040_si_001.pdf [file pg2c00040_si_001.pdf]

Supplementary Information
for:
Counting the acid sites in a commercial ZSM-5 zeolite catalyst

Andrea Zachariou[a,b] (http://orcid.org/0000-0002-9083-787X),

Alexander P. Hawkins[a,b] (http://orcid.org/0000-0002-5947-6631),

Russell F. Howe[c] (http://orcid.org/0000-0003-2462-8962),

Janet M. S. Skakle[c,d], (https://orcid.org/0000-0001-5249-5306)

Nathan Barrow[e],

Paul Collier[e],

Daniel W. Nye[f]

Ronald I. Smith[f] (https://orcid.org/0000-0002-4990-1307)

Gavin B. G. Stenning[f]

Stewart F. Parker[a,b,f,] (http://orcid.org/0000-0002-3228-2570, stewart.parker@stfc.ac.uk)

David Lennon[a*] (http://orcid.org/0000-0001-8397-0528),

[a] *School of Chemistry, University of Glasgow, Joseph Black Building, Glasgow, G12 8QQ*
[b] *UK Catalysis Hub, Research Complex at Harwell, STFC Rutherford Appleton Laboratory, Chilton, Oxon, OX11 0FA, UK*
[c] *Department of Chemistry, University of Aberdeen, Aberdeen, AB24 3UE, UK*
[d] *Department of Physics, University of Aberdeen, Aberdeen, AB24 3UE, UK*
[e] *Johnson Matthey Technology Centre, Blounts Court, Sonning Common, Reading, RG4 9NH, UK*
[f] *ISIS Facility, STFC Rutherford Appleton Laboratory, Chilton, Oxon, OX11 0QX, UK.*

* Email: David.Lennon@glasgow.ac.uk

Keywords: HZSM-5, Inelastic neutron scattering (INS), DRIFTS, NMR, SEM, ammonia chemisorption

Surface area and volume measurements

BET measurements produced the isotherm shown in Fig. S1 (upper part). The hysteresis visible between the adsorption and desorption isotherms is due to condensation of the nitrogen in mesopore-sized gaps between the zeolite crystals, evidenced by the lack of a sharp onset in the hysteresis region and its location at high relative pressures. The formation of multiple layers and subsequent condensation is also responsible for the fact that the isotherm does not attain a plateau value at high relative pressures [1]. BET analysis of this isotherm shows the sample to have a total surface area of 370 ± 11 m^2g^{-1} as an average and standard deviation based on three measurements. It should be noted that the adsorbate gas used was nitrogen which has a cross-sectional area of 16.2 $Å^2$. This is close to the cross-sectional areas of the zeolite channels which are 23.3 $Å^2$ for the straight channels and 18.0 $Å^2$ for the sinusoidal channels. This therefore means that these values represent a 'nitrogen-accessible' surface area and micropore volume which can be used for relative comparisons between samples but which is lower than the true properties of the zeolite.

The *t*-plot, Fig. 3 (lower part) shows an inflection in the curve located at a statistical thickness value of *ca.* 4.5 Å, which indicates the presence of two types of pore sizes. The data points located below this value correspond to adsorption in the micropores: *t*-plot analysis cannot be used below a statistical thickness of 3.5 Å due to the size of the nitrogen molecule, but the linear trend of the data available suggests a micropore surface area of 248 ± 9 m^2g^{-1} with a volume of 0.101 ± 0.003 cm^3g^{-1}. The points above this value, but below the region of hysteresis in Fig. 3 (lower part), correspond to adsorption in mesopores of 9 – 10 Å in diameter, formed by defects in the MFI structure. These have a surface area of 72 ± 2 m^2g^{-1} and a volume of 0.032 ± 0.001 cm^3g^{-1}. The remaining surface area in the total BET measurement, amounting to 50 m^2g^{-1}, is the external surface area, which comprises both adsorption on the exterior surface of the crystals and condensation in the intercrystalline voids. These properties closely match those reported for commercially produced ZSM-5 zeolites in the literature [2].

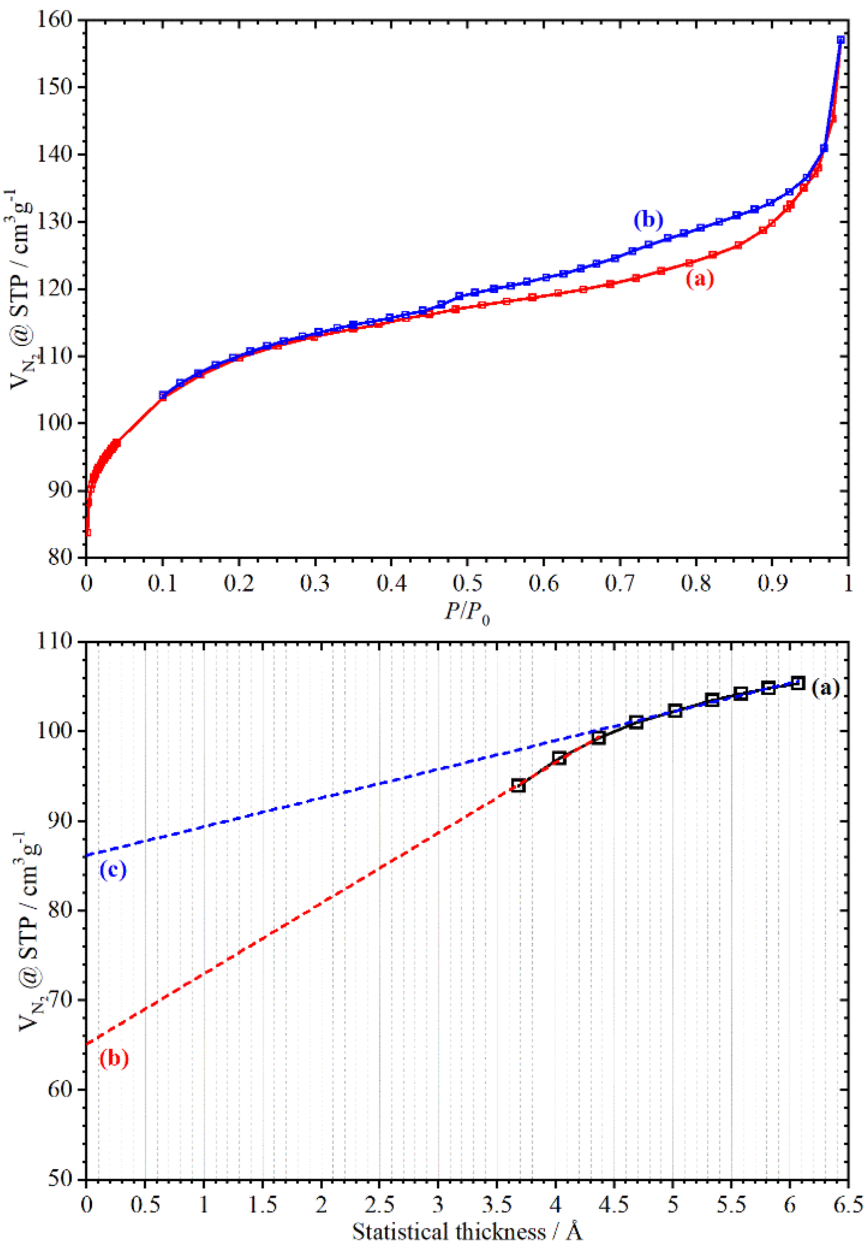

Fig. S1 Top: adsorption (a) and desorption (b) isotherms for N_2 on HZSM-5, showing hysteresis due to sample microporosity. Bottom: statistical thickness plot for the adsorption isotherm of nitrogen in ZSM-5 (a) showing the linear fits used to derive the micropore (b) and mesopore (c) volumes according to the method of de Boer [12].

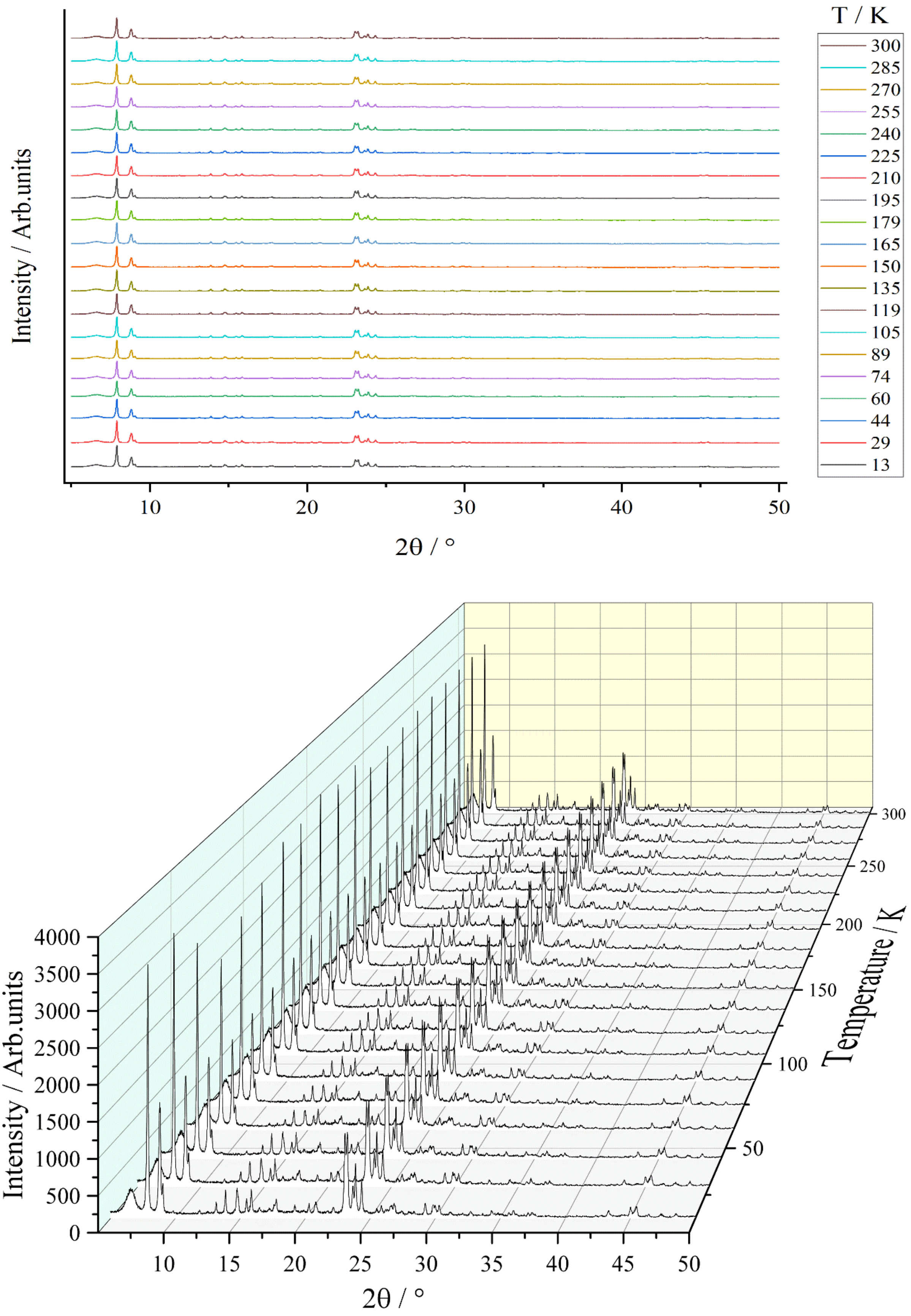

Fig. S2 Variable temperature (13 – 300 K) powder XRD data for HZSM-5 displayed in offset mode (top) and as a waterfall plot (bottom).

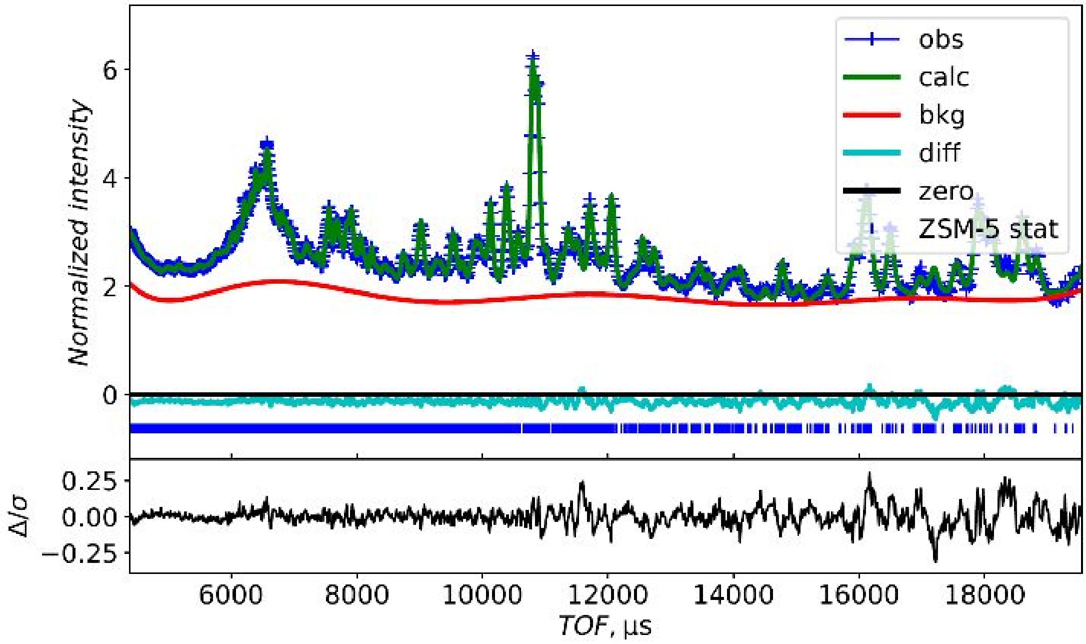

Fig. S3 Fitted room temperature Polaris bank 4 powder neutron diffraction pattern from ZSM-5 using the *Pnma* orthorhombic structure.

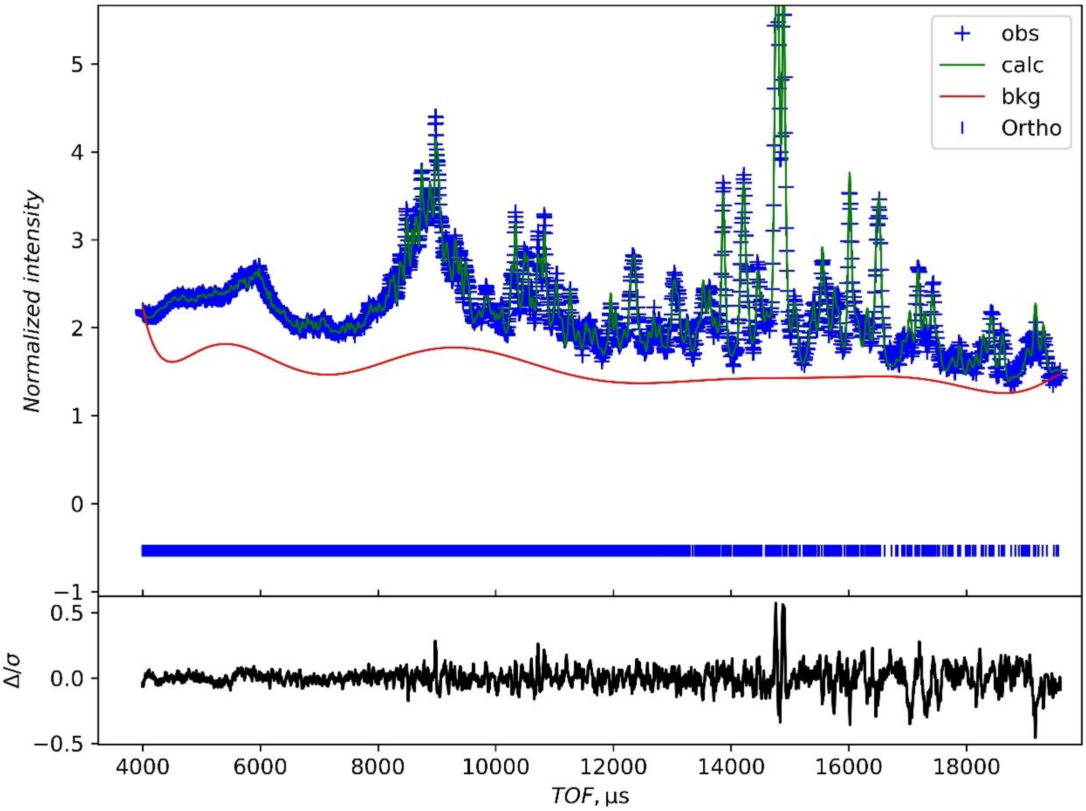

Fig. S4 Fitted room temperature Polaris bank 5 powder neutron diffraction pattern from HZSM-5 using the *Pnma* orthorhombic structure.

Table S1

HZSM-5 orthorhombic model in *Pnma.*

a = 20.1437(9) b = 19.9613(9) c = 13.4268(7) Å V = 5398.9(5) Å^3

Bank 4: wR = 1.82%, R = 2.19%, Rf = 1.25% on 1570 observations. DW = 0.337

Bank 5: wR = = 1.71%, R = 2.06 %, Rf = 1.19% on 3180 observations. DW = 0.346

Combined: wR = 1.77%, GOF = 1.73, 4750 observations.

Atom	x/a	y/b	z.c	Uiso
Si0	0.0708(19)	0.6293(21)	0.3225(28)	0.036(9)
Si1	0.0732(11)	0.0307(12)	0.3244(20)	0.016(5)
Si2	0.0699(15)	0.5562(19)	0.6725(20)	0.028(8)
Si3	0.0703(11)	0.1812(11)	0.6784(16)	0.022(4)
Si4	0.1311(12)	0.6707(12)	0.5326(18)	0.018(6)
Si5	0.1177(12)	0.0600(13)	0.5265(16)	0.002(5)
Si6	0.1881(17)	0.6698(14)	0.1782(21)	0.013(7)
Si7	0.1897(14)	0.5292(12)	0.8241(21)	0.016(5)
Si8	0.1882(16)	0.0590(15)	0.1825(18)	0.012(5)
Si9	0.1875(16)	0.1250(15)	0.7984(19)	0.015(6)
Si10	0.2181(13)	0.5646(16)	0.0296(19)	0.015(6)
Si11	0.2279(13)	0.1736(13)	0.0340(17)	0.013(5)
O12	0.0009(11)	0.5506(11)	0.7157(14)	0.016(5)
O13	0.0054(9)	0.1382(9)	0.7087(15)	0.016(4)
O14	0.0823(10)	0.6280(10)	0.6173(13)	0.017(4)
O15	0.0906(9)	0.6647(9)	0.4288(14)	0.006(5)
O16	0.0962(8)	0.0700(10)	0.4073(15)	0.008(4)
O17	0.0792(7)	0.5509(9)	0.3458(11)	0.0019(26)
O18	0.0920(9)	0.0045(10)	0.5902(14)	0.0065(31)
O19	0.0980(8)	0.1340(10)	0.5722(12)	0.0028(31)
O20	0.1158(7)	0.6571(7)	0.2368(11)	0.0039(30)
O21	0.1192(9)	0.0470(9)	0.2226(14)	0.011(5)
O22	0.1211(9)	0.5528(12)	0.7658(13)	0.012(4)
O23	0.1308(9)	0.1597(8)	0.7561(13)	0.001(3)
O24	0.1871(9)	0.5564(9)	0.9278(12)	0.006(4)
O25	0.1912(10)	0.0499(10)	0.8088(12)	0.015(4)
O26	0.1873(11)	0.1465(8)	0.9325(12)	0.009(4)
O27	0.1978(10)	0.6541(8)	0.5341(12)	0.003(3)
O28	0.1846(12)	0.1244(10)	0.1173(14)	0.012(5)
O29	0.1951(11)	0.0600(12)	0.5192(12)	0.016(4)
O30	0.2003(9)	0.6330(8)	0.0885(10)	0.0021(27)
O31	0.2030(9)	-0.0008(8)	0.1012(12)	0.007(3)
O32	0.2414(9)	0.6562(9)	0.2765(12)	0.006(4)
O33	0.2493(8)	0.0642(10)	0.2546(13)	0.006(4)
O34	0.0724(17)	0.25	0.6385(20)	0.022(7)
O35	0.1130(11)	0.75	0.5791(16)	0.010(4)
O36	0.1894(13)	0.75	0.1627(16)	0.018(4)
O37	0.2110(11)	0.25	0.0561(16)	0.010(4)

Table S2

H- ZSM5 monoclinic model in $P2_1/n$.

a = 19.9534(7) b = 20.1334(7) c = 13.4214(7) Å β = 89.953(8) V = 5391.8(4) $Å^3$

Bank 4: wR = 1.22%, R = 1.56%, Rf = 0.94% on 1607 observations.

Bank 5: wR = = 1.47%, R = 1.71%, Rf = 1.23% on 3214 observations.

Combined: wR = 1.35%, GOF = 1.43, 4821 observations.

Si (Uiso) = 0.0126(8), O (Uiso) = 0.0043(2)

Atom	x/a	y/b	z/c
Si1	0.0542(16)	0.4254(15)	-0.3264(21)
Si2	0.0286(14)	0.3107(16)	-0.178(2)
Si3	0.0612(15)	0.2766(11)	0.0405(20)
Si4	0.0606(16)	0.1224(12)	0.027(2)
Si5	0.0253(18)	0.0758(18)	-0.184(4)
Si6	0.0532(15)	0.1926(15)	-0.322(2)
Si7	-0.1772(16)	0.4279(16)	-0.325(2)
Si8	-0.1317(16)	0.3085(15)	-0.189(2)
Si9	-0.1776(16)	0.2748(16)	0.031(2)
Si10	-0.1802(16)	0.1179(14)	0.0337(19)
Si11	-0.1307(14)	0.0717(13)	-0.1770(19)
Si12	-0.1677(12)	0.1851(13)	-0.3182(20)
Si13	0.4393(14)	0.4271(14)	-0.3357(18)
Si14	0.4687(12)	0.3110(14)	-0.1886(18)
Si15	0.4352(14)	0.2737(14)	0.0284(20)
Si16	0.4380(14)	0.1177(14)	0.0257(18)
Si17	0.4697(13)	0.0702(13)	-0.1908(19)
Si18	0.4368(13)	0.1880(12)	-0.3219(21)
Si19	0.6704(13)	0.4251(13)	-0.329(2)
Si20	0.6276(13)	0.3108(14)	-0.1826(19)
Si21	0.6686(14)	0.2762(13)	0.0311(19)
Si22	0.6723(14)	0.1236(13)	0.0341(18)

Si23	0.6306(14)	0.0744(15)	-0.1812(18)
Si24	0.6750(12)	0.1905(13)	-0.3143(18)
O1	0.0504(18)	0.3812(15)	-0.2266(21)
O2	0.0546(18)	0.3112(17)	-0.0663(18)
O3	0.0478(18)	0.2014(3)	0.0372(18)
O4	0.0642(17)	0.1091(14)	-0.0922(20)
O5	0.0392(15)	0.1231(14)	-0.272(2)
O6	0.0573(19)	0.2507(15)	-0.237(2)
O7	-0.1585(16)	0.3752(16)	-0.239(3)
O8	-0.1635(14)	0.2958(16)	-0.0838(19)
O9	-0.1647(12)	0.1965(14)	0.032(2)
O10	-0.1728(15)	0.0914(17)	-0.0792(19)
O11	-0.1457(16)	0.1142(14)	-0.2748(21)
O12	-0.1395(18)	0.2424(14)	-0.2474(21)
O13	-0.0515(13)	0.3209(13)	-0.1689(21)
O14	-0.0520(14)	0.0750(15)	-0.150(2)
O15	0.1205(16)	0.4159(17)	-0.394(2)
O16	-0.0055(17)	0.3990(16)	-0.394(2)
O17	-0.1314(15)	0.4061(18)	-0.426(2)
O18	0.1270(14)	0.1957(20)	-0.3725(19)
O19	0.0003(16)	0.2097(14)	-0.407(2)
O20	-0.1317(14)	0.1966(17)	-0.423(2)
O21	0.0481(18)	0.0015(14)	-0.201(2)
O22	-0.1400(16)	-0.0053(12)	-0.205(2)
O23	-0.2524(14)	0.4228(16)	-0.3589(19)
O24	-0.2455(11)	0.1997(15)	-0.3374(17)
O25	-0.2550(13)	0.2942(13)	0.0553(16)
O26	-0.2543(13)	0.1107(14)	0.0781(20)
O27	0.4390(14)	0.3762(15)	-0.241(2)
O28	0.4465(17)	0.3156(17)	-0.0753(17)

O29	0.4288(13)	0.1958(14)	0.0032(19)
O30	0.4394(18)	0.0860(14)	-0.0838(19)
O31	0.4390(18)	0.1140(14)	-0.2781(20)
O32	0.4337(17)	0.2493(15)	-0.246(2)
O33	0.6556(19)	0.3705(15)	-0.245(2)
O34	0.6437(15)	0.3167(14)	-0.0655(17)
O35	0.6472(15)	0.1993(13)	0.027(2)
O36	0.6553(17)	0.0872(17)	-0.0682(19)
O37	0.6677(15)	0.1174(14)	-0.266(2)
O38	0.6598(15)	0.2444(15)	-0.226(2)
O39	0.5483(12)	0.3104(17)	-0.1996(20)
O40	0.5503(13)	0.0782(16)	-0.185(2)
O41	0.3694(16)	0.4211(17)	-0.393(2)
O42	0.4976(14)	0.4109(16)	-0.4170(21)
O43	0.6337(16)	0.4000(19)	-0.431(2)
O44	0.3721(14)	0.1892(19)	-0.391(2)
O45	0.4978(15)	0.1978(18)	-0.3950(20)
O46	0.6317(15)	0.2070(17)	-0.410(2)
O47	0.4537(18)	-0.0053(12)	-0.221(2)
O48	0.6530(18)	-0.0004(15)	-0.205(2)

References

[1] K.S.W. Sing, Reporting physisorption data for gas/solid systems with special reference to the determination of surface area and porosity (Recommendations 1984), Pure Appl. Chem. 57 (4) (1985) 603-619. https://doi.org/10.1351/pac198557040603.

[2] L. Emdadi, Y. Wu, G. Zhu, C.-C. Chang, W. Fan, T. Pham, R.F. Lobo, D. Liu, Dual Template Synthesis of meso- and microporous MFI zeolite nanosheet assemblies with tailored activity in catalytic reactions. Chem. Mater. 26 (3) 2014, 1345-1355. https://doi.org/10.1021/cm401119d.

[3] G. Artioli, C. Lamberti, G.L. Marra, Neutron powder diffraction study of orthorhombic and monoclinic defective silicalite, Acta Cryst. B56 (1999) 2-10. https://doi.org/10.1107/S0108768199008927.
